# Adipose Tissue-Derived Mesenchymal Stem Cells Exert In Vitro Immunomodulatory and Beta Cell Protective Functions in Streptozotocin-Induced Diabetic Mice Model

**DOI:** 10.1155/2015/878535

**Published:** 2015-03-29

**Authors:** Hossein Rahavi, Seyed Mahmoud Hashemi, Masoud Soleimani, Jamal Mohammadi, Nader Tajik

**Affiliations:** ^1^Division of Transplant Immunology and Immunogenetics, Immunology Research Center (IRC), Iran University of Medical Sciences, Tehran, Iran; ^2^Department of Immunology, School of Medicine, Shahid Beheshti University of Medical Sciences, Tehran, Iran; ^3^Department of Stem Cell Biology, Stem Cell Technology Research Center, Tehran, Iran; ^4^Department of Hematology, School of Medical Sciences, Tarbiat Modares University, Tehran, Iran; ^5^Department of Immunology, School of Medicine, Tehran University of Medical Sciences, Tehran, Iran

## Abstract

Regenerative and immunomodulatory properties of mesenchymal stem cells (MSCs) might be applied for type 1 diabetes mellitus (T1DM) treatment. Thus, we proposed in vitro assessment of adipose tissue-derived MSCs (AT-MSCs) immunomodulation on autoimmune response along with beta cell protection in streptozotocin- (STZ-) induced diabetic C57BL/6 mice model. MSCs were extracted from abdominal adipose tissue of normal mice and cultured to proliferate. Diabetic mice were prepared by administration of multiple low-doses of streptozotocin. Pancreatic islets were isolated from normal mice and splenocytes prepared from normal and diabetic mice. Proliferation, cytokine production, and insulin secretion assays were performed in coculture experiments. AT-MSCs inhibited splenocytes proliferative response to specific (islet lysate) and nonspecific (PHA) triggers in a dose-dependent manner (*P* < 0.05). Decreased production of proinflammatory cytokines, such as IFN-*γ*, IL-2, and IL-17, and increased secretion of regulatory cytokines such as TGF-*β*, IL-4, IL-10, and IL-13 by stimulated splenocytes were also shown in response to islet lysate or PHA stimulants (*P* < 0.05). Finally, we demonstrated that AT-MSCs could effectively sustain viability as well as insulin secretion potential of pancreatic islets in the presence of reactive splenocytes (*P* < 0.05). In conclusion, it seems that MSCs may provide a new horizon for T1DM cell therapy and islet transplantation in the future.

## 1. Introduction

Type 1 diabetes mellitus (T1DM) is identified by the progressive autoimmune destruction of pancreatic beta cells, which results in a dramatic decrease of insulin production and consequent metabolic complications. Transplantation of human cadaveric pancreas or allogeneic islet cells could be considered therapeutic in this condition. However, the scarcity of cadaveric pancreas donors necessitates search for alternative cell sources [1]. In addition, replacement of the beta cell deficit along with regulation of autoimmune response to cells that express insulin is crucial for a T1DM definitive cure. Thus, in recent years, the usage of cell sources that modulate immune system along with islet cell replacement has received much attention [2].

Mesenchymal stem cells (MSCs) represent a rare heterogeneous subset of multipotent stromal cells localized in many different adult and fetal tissues. They have self-renewal and multidifferentiation capacity that can give rise to diverse lineages of mesenchymal origin, including osteoblasts, adipocytes, and chondrocytes, and have also shown their potential for differentiating into nonmesodermal origin cells [3]. Due to these properties, MSCs might be useful in tissue regeneration and cell-based therapies [4]. Although multipotent MSCs are usually isolated from bone marrow (BM), more recently, adipose tissue-derived MSCs (AT-MSCs) due to more quantities, simple accessibility, and also the better immunomodulatory properties were represented as another alternative source for MSCs [5, 6]. Numerous recent studies indicated that MSCs possess immunomodulatory or immunosuppressive effects both in vitro and in vivo on several immune cells, not only T lymphocytes but also on B lymphocytes, dendritic cells (DCs), and NK cells [7]. In vitro studies have identified that the immunomodulatory function of MSCs can be addressed by both cell-cell contact [8] and soluble factors [9, 10].

MSCs can inhibit immune cells proliferation, reduce inflammatory cytokines secretion, and alter immune cell types to regulatory clones. They exert immune regulation by the secretion of anti-inflammatory factors, such as interleukin-10 (IL-10) [11], transforming growth factor-*β* (TGF-*β*), indoleamine 2,3-dioxygenase (IDO) [12, 13], nitric oxide [9], prostaglandin-E2 (PGE-2) [14], and human leukocyte antigen-G (HLA-G) [15]. In addition, MSCs induce cell cycle arrest and apoptosis of T lymphocytes [11, 16] and cause lymphocytes to secrete regulatory cytokines, especially IL-10 [11].

Mesenchymal stem cells due to their low immunogenicity and immunomodulatory properties as well as high degree of differentiation and proliferation potential might be useful in inhibiting the autoimmunity and regenerating the insulin-secreting cells [17]. Furthermore, many studies declared that the regenerative role of MSCs could be mediated by protective effects on functional islet cells and also differentiation potency to insulin-producing cells in vivo and in vitro [18]. The ideal use of MSCs in autoimmune diabetes regenerative therapy can only be obtained after we learn their immunomodulatory characteristics in detail. The aim of this research was to evaluate the immunomodulatory effects of adipose-derived MSCs on autoimmune response besides islet protective function in streptozotocin- (STZ-) induced diabetic mice model. This study may provide some basic information regarding application of human MSCs in treating type 1 diabetes.

## 2. Materials and Methods

### 2.1. Mice and Induction of Experimental Type 1 Diabetes

Female inbred C57BL/6 mice (6–8 weeks old) were purchased from the Pasteur Institute, Tehran, Iran. They were given sterilized water and autoclaved standard mouse chow throughout the study. Diabetes was induced in the mice by intraperitoneal injection of multiple low-doses (50 mg/kg, 4 consecutive days) of streptozotocin (STZ) (Sigma, USA). STZ was solubilized in the sodium citrate buffer, pH 4.5, and injected within 10 min of preparation.

### 2.2. Isolation and Expansion of Adipose Tissue-Derived Mesenchymal Stem Cell (AT-MSCs)

Abdominal adipose tissue was taken from C57Bl/6 mice after sacrificing, washed 3 times with phosphate-buffered saline (PBS), and minced. Extracellular matrix was digested with 0.075% type I collagenase (37°C and 5% CO_2_ for 30 min) and centrifuged at 500 g for 5 min; then the pellet was cultured in high glucose Dulbecco's modified Eagle's medium (DMEM, GIBCO) containing 10% fetal bovine serum (FBS, GIBCO), 2 mM L-glutamine, penicillin, and streptomycin (all from Invitrogen) as MSCs culture media and incubated at 37°C in 5% CO_2_. After 48 h, nonadherent cells were removed and fresh media were added. When adherent cells were confluent, they were trypsinized, harvested, and expanded. All the experiments were performed using AT-MSCs at passage 3.

### 2.3. Immunophenotype Analyses

The cell surface markers on AT-MSCs were assessed using monoclonal antibodies against mouse CD73, CD105, CD29, CD90, CD31, CD11b, CD45, and CD34 (all from eBioscience). The AT-MSCs at passage 3 were detached with 0.25% trypsin/EDTA and resuspended to 5 × 10^5^ cells in PBS. The cells were incubated with the specific or isotype control antibodies (mouse IgG1-FITC and mouse IgG1-PE, eBioscience) in 100 *μ*L of 3% bovine serum albumin (BSA, Sigma) in PBS for 1 hour at 4°C. The cells were then fixed with 1% paraformaldehyde (Sigma) and analyzed using a FACS Calibur flow cytometer (BD Biosciences, San Diego, CA) and Cyflogic software (CyFlo Ltd.).

### 2.4. Multilineage Differentiation

The AT-MSCs at passage 3 were analyzed for their ability to differentiate into osteoblast, adipocyte, and chondrocyte. For osteogenic differentiation, cells were cultured in medium containing 10 mM beta-glycerophosphate (Merck), 50 mg/mL ascorbic acid biphosphate (Sigma), and 100 nM dexamethasone (Sigma). After 3-week induction, cells were stained with alizarin red to assess mineralization. For adipogenic differentiation, cells were cultured in the presence of 250 nM dexamethasone (Sigma), 0.5 mM 3-isobutyl-1-methylxanthine (Sigma), 5 mM insulin (Sigma), and 100 mM indomethacin (Sigma). After 3 weeks oil red staining was used to determine the accumulation of oil droplets in the cytoplasm. For differentiation to chondrocytes, 1 × 10^4^ cells were centrifuged to form a pelleted micromass and then treated with TGF-beta (10 ng/mL; Merck), ascorbic acid biphosphate (50 *μ*g/Ml), dexamethasone (10 *μ*M), and insulin transferrin selenium (ITS) (50 *μ*g/Ml; Sigma) for 3 weeks. Chondrocyte differentiation was assessed by alcian blue staining on sections obtained from micromasses.

### 2.5. Isolation of Pancreatic Islets

Pancreatic islets were isolated by a modified collagenase digestion method [19]. Briefly, pancreas was excised from C57BL/6 mice under sterile conditions, inflated by collagenase type XI (1 mg/mL; Sigma) for few moments, and minced into small pieces. The enzymatic digestion of the pancreatic tissue was fulfilled by collagenase type XI in 37°C water bath for 20 min with interim agitation. Then, digested contents were filtered through 500 and 100 *μ*m cell strainers, respectively, to capture the islets and allow the small exocrine cells to pass. The enriched islets were hand picked using a sampler under a stereomicroscope. The purified islets were counted and characterized by dithizone (DTZ) staining. To evaluate islet cell function, islets (*n* = 10) were stimulated in RPMI-1640 culture solution with low glucose (5.6 mmol/L) and incubated for 4 hours at 37°C for detection of the total levels of insulin in the culture solution. The RPMI-1640 culture solution was then switched to high glucose (16.7 mmol/L) and culture performed under the same condition (37°C, 4 hours) for insulin determination. Islet cells lysate was prepared by freezing and thawing 10 islets in 0.5 mL of RPMI-1640 medium supplemented with 10% FBS (assuming one islet contains 1000 single cells) [20].

### 2.6. Splenocytes Proliferation Assay

The spleen was removed from the normal and diabetic mice and placed in cold RPMI-1640 media. Splenocytes were extracted using a 5 mL syringe with a 23 G needle. RBC was lysed with ammonium chloride solution and cells were washed twice. Cell suspensions were washed in cold RPMI-1640 media and counted and viability was assessed by 0.2% trypan blue. RPMI-1640 supplemented with 10% heat inactivated FBS, 100 mg/mL streptomycin, 100 units/mL penicillin, 2 mM L-glutamine, and 10 mM HEPES was used as splenocyte culture medium. In proliferation assay, normal and diabetic splenocytes were cocultured with AT-MSCs in the MSCs culture medium mixed 1 : 1 with fresh splenocyte culture medium (mixed culture medium). Prior to final plating, optimized concentration of splenocytes with or without phytohemagglutinin (PHA, GIBCO) was determined at dilutions of 1, 2, 3, 4, and 5 × 10^5^ cells in 96-well plate by MTT assay. Final density of splenocytes was adjusted to 2.5 × 10^5^ cells per well for coculture with AT-MSCs. AT-MSCs at passage 3 were harvested and adjusted to 2 × 10^2^/mL, 1 × 10^3^/mL, and 5 × 10^3^/mL in MSCs culture medium containing 10% FBS. A 100 *μ*L suspension of AT-MSCs was plated into 96-well plates and incubated for 24 h at 37°C. After the AT-MSCs reached 70% confluence, the medium was removed, and 100 *μ*L of fresh medium containing 5 *μ*L of mitomycin-C (1 *μ*g/*μ*L; Sigma) was added for 1 h at 37°C to mitotically inactivate the AT-MSCs. After that medium was removed and inactivated AT-MSCs were washed twice with PBS. AT-MSCs were resuspended in 100 *μ*L of mixed culture medium, cocultured with 2.5 × 10^5^ splenocytes, and stimulated by 5 *μ*g/mL PHA (10 *μ*L) or 10 *μ*L of islet cells lysate in control and test groups, respectively, for 48 h at 37°C. The test and control groups in proliferation experiments were designed as in Table 1 representing the content of each well in culture plate. Three ratios of AT-MSCs to splenocytes were used: 1 : 50, 1 : 250, and 1 : 1250. The suppressive effect of AT-MSCs on splenocytes proliferation in the absence and presence of PHA (as nonspecific stimulator) and islet cells lysate (as specific stimulator) was determined by MTT [3-(4,5-dimethylthiazol-2-yl)-2,5-diphenyl tetrazolium bromide] assay. The cells, cultured in a 96-well plate, were incubated for 4 h in the presence of MTT (5 mg/mL; Sigma) followed by addition of 0.1 mL dimethyl sulfoxide (DMSO). The formazan crystals were dissolved and the absorbance was read at 570 nm by ELISA reader. The proliferation index was calculated using the following formula:

(1) Proliferation  index=ODMSCs+Splenocytes±stimulator−ODMSCsODSplenocytes.

In order to examine the proliferation of splenocytes (in the presence of AT-MSCs), AT-MSCs monoculture was used as blank to subtract background absorbance of AT-MSCs. To provide the optimum condition, the MTT assay was repeated several times with different ratios of AT-MSCs to splenocytes, various concentrations of PHA, and also different periods of coculture incubation.

### 2.7. Cytokine Production Assay

After being trypsinized, AT-MSCs were adjusted to 2.5 × 10^4^/mL and plated (1 mL) in 24-well plates. After the AT-MSCs reached 70% confluence, 10 *μ*L of mitomycin-C (1 *μ*g/*μ*L) was added into each well. After 1 h at 37°C and 5% CO_2_, the medium was removed and cells were washed twice with PBS. Then 12.5 × 10^5^ splenocytes in 1 mL of mixed culture medium were added and cocultured in the presence of 50 *μ*L of PHA or 50 *μ*L islet lysate for 72 h at 37°C. The groups were similar to those described for proliferation. The supernatants of each group were collected and evaluated by Multi-Analyte ELISArray Kit (MEM-003A, SABiosciences) for TGF-*β*, IL-10, IL-4, and IL-13 as regulatory cytokines and IFN-*γ*, IL-2, and IL-17A as inflammatory cytokines. In order to examine cytokine production by splenocytes, AT-MSCs were used as blank to subtract background absorbance of the cytokines produced by AT-MSCs.

### 2.8. Insulin Secretion Assay

AT-MSCs at passage 3 were seeded in 96-well plates at density of 5 × 10^3^ cells/well. After reaching the ideal confluency, the medium was removed and cells were washed twice with PBS. Then 2.5 × 10^5^ normal and diabetic splenocytes were added and cocultured with 10 freshly isolated islets (assuming one islet contains 1000 single cells) in 100 *μ*L of mixed culture medium (low glucose RPMI-1640 and high glucose DMEM) supplemented with 10% FBS for 24 h at 37°C. After incubation period, the medium was removed slowly; then the islets were challenged with 100 *μ*L stimulatory medium containing high glucose DMEM supplemented with 5 mmol/L theophylline for a 10 min period. The supernatants of each group were collected and evaluated by mouse insulin ELISA kit (EZRMI-13k, Millipore). The groups were designed as follows: islet cells (positive control), islet cells + normal splenocytes, islet cells + diabetic splenocytes, islet cells + normal splenocytes + MSCs, islet cells + diabetic splenocytes + MSCs, and splenocytes (negative control).

### 2.9. Statistical Analysis

All data are expressed as the mean ± SD. Statistical analysis was done using the one-way analysis of variance (ANOVA) to compare results. Values of *P* < 0.05 were considered to be statistically significant.

## 3. Results

### 3.1. Induction of Experimental Diabetes

In this study, diabetic mice model was developed by administration of multiple low-doses of STZ. The blood glucose levels of ≥300 mg/dL were monitored within 1 week of STZ treatment. In addition, the insulin levels of 4.95 ± 0.52 ng/dL in normal mice decreased to <0.5 ng/dL in diabetic mice and pancreatic islets destruction was confirmed by histopathological examination.

### 3.2. Characterization of AT-MSCs

MSCs seeded to the culture flasks sparsely and the cells displayed a fibroblast-like morphology during the early days of incubation. After 6–8 days, the cells gradually grew to form small colonies that were termed colony-forming units. As growth continued, colonies gradually expanded in size and the adjacent ones interconnected with each other. These primary cells reached monolayer confluence after plating for 10–12 days in their first passages. In later passages, MSCs appeared to adopt a uniform fibroblast-like morphology.

AT-MSCs at passage 3 were evaluated for the expression of specific cell surface markers. The cells lacked CD11b, CD34, CD45, and CD31 whereas they were all positive for CD73, CD90, CD105, and CD29 expression. Moreover, AT-MSCs were able to differentiate toward osteogenic, adipogenic, and chondrogenic lineages. After 21 days, osteogenesis of AT-MSCs was demonstrated by mineralization of the extracellular matrix with alizarin red staining. Additionally, lipid droplets were detectable by oil red O staining after three weeks of adipocytic induction. After 21 days of induction, chondrogenic differentiation of AT-MSCs was achieved. More than 80% of all cells stained positively with alcian blue showed the glucose amino glycan (GAG) biosynthesis in the cell pellets (a figure illustrating AT-MSCs characterization has been presented as supplementary data in Supplementary Material available online at http://dx.doi.org/10.1155/2015/878535).

### 3.3. Isolation of Pancreatic Islets

The perfusion of pancreas through common bile duct is a complicated manipulation and needs professional experience. Moreover, purification of islets by Ficoll gradient method could have toxic side effects on islet cells. In this study, we modified the procedure by excluding the in situ pancreas perfusion and Ficoll gradient steps. The islet count showed that the efficiency of isolation varied depending on animal age and strain. The yield of isolation was between 30 and 50 islets for each pancreas. However, the duration of whole procedure decreased to 30–40 min for each mouse. The purified islets showed intact morphology with smooth surface and were free of exocrine cells as confirmed with DTZ staining. The viability of islets was greater than 95% as determined by trypan blue.

### 3.4. AT-MSCs Reduce In Vitro Proliferation of Mitogen-Stimulated Splenocytes

This experiment was designed to investigate whether AT-MSCs could inhibit proliferation of splenocytes that were triggered by PHA as a nonspecific stimulator. Firstly, to assess which concentration of splenocytes exerts optimized proliferation, we cultured resting cells at dilutions of 1, 2, 3, 4, and 5 × 10^5^ in the absence or presence of PHA. After drawing standard curve, the final density of normal splenocytes was adjusted to 2.5 × 10^5^ cells for coculture with AT-MSCs.

**Figure 1:**
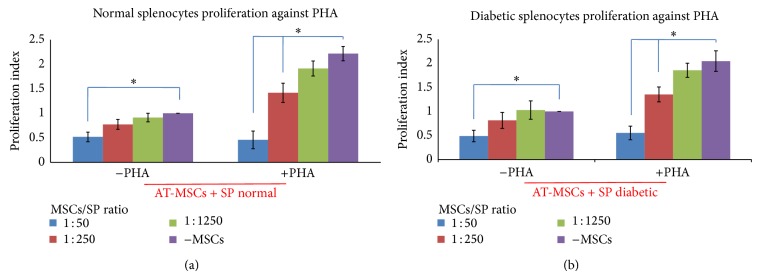
Proliferation index of splenocytes from normal (a) and diabetic (b) mice cocultured with AT-MSCs in presence of PHA as nonspecific stimulator. The resting splenocytes showed a sharp proliferation response in the presence of PHA. AT-MSCs caused cell death in some resting splenocytes in the absence of mitogen (proliferation index < 1.0). AT-MSCs could suppress normal and diabetic splenocytes proliferation in response to PHA in a dose-dependent manner, where greater concentration of AT-MSCs induced cell death in some splenocytes (proliferation index < 1.0). Each bar represents the average of five independent experiments. ∗ indicates significant difference between groups (*P* < 0.05). PHA: phytohemagglutinin; SP: splenocytes.

To evaluate antiproliferative effect of AT-MSCs, they were added to PHA-stimulated splenocytes cultures with a ratio of AT-MSCs to splenocytes of 1 : 50, 1 : 250, and 1 : 1250 and cell proliferation was determined. As expected, the resting splenocytes showed a strong proliferation response in the presence of PHA (Figure 1; *P* < 0.05), whereas addition of AT-MSC to PHA-stimulated splenocytes resulted in a significant reduction of splenocytes proliferation in a number-dependent manner (Figure 1; *P* < 0.05). Higher inhibition of splenocytes proliferation was shown at the lowest dilution tested (1 : 50). The results also indicated that AT-MSCs could induce cell death of some splenocytes in the absence of mitogen (Figure 1; *P* < 0.05). The greater cell death of splenocytes seemed to occur at the highest density of AT-MSCs (1 : 50). In mitogen-induced proliferation experiment, no significant difference was detected between normal and diabetic splenocytes in the pattern of reduced proliferation and response to mitogen.

### 3.5. AT-MSCs Exert Antiproliferative Effect on Splenocytes Stimulated with Islet Cells Lysate

**Figure 2:**
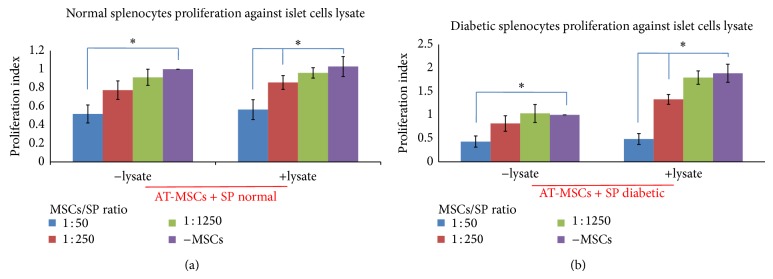
Proliferation index of splenocytes from normal (a) and diabetic (b) mice cocultured with AT-MSCs in presence of islet cells lysate as specific stimulator. Diabetic splenocytes showed a sharp proliferation in the presence of lysate, in which normal splenocytes remained nonproliferated. AT-MSCs caused cell death of some resting splenocytes in the absence of lysate (proliferation index < 1.0). AT-MSCs could suppress diabetic splenocytes proliferation in response to lysate in a dose-dependent manner, where higher concentration of AT-MSCs induced cell death in some splenocytes (proliferation index < 1.0). Each bar represents the average of five independent experiments. ∗ indicates significant difference between groups (*P* < 0.05). Lysate: pancreatic islet cells lysate; SP: splenocytes.

After confirming that AT-MSCs could inhibit proliferation of mitogen-stimulated splenocytes, we then investigated whether AT-MSCs could inhibit proliferation of splenocytes triggered by specific stimulator. AT-MSCs were cocultured with islet lysate-stimulated splenocytes as previously described and cell proliferation was assessed. The normal splenocytes did not proliferate upon addition of islet lysate. In contrast, diabetic cells showed a significant proliferation (Figure 2; *P* < 0.05). Again, the dose-dependent effect of AT-MSCs on diabetic splenocytes proliferation inhibition was observed in presence of islet lysate (Figure 2; *P* < 0.05). At the lowest dilution tested (1 : 50), more significant inhibition of splenocytes proliferation was observed. The diabetic splenocytes did not significantly proliferate in the absence of islet lysate. However, the results demonstrated that AT-MSCs could induce cell death in some of these splenocytes (Figure 2; *P* < 0.05).

### 3.6. Effect of AT-MSCs on Cytokines Produced by Splenocytes

**Figure 3:**
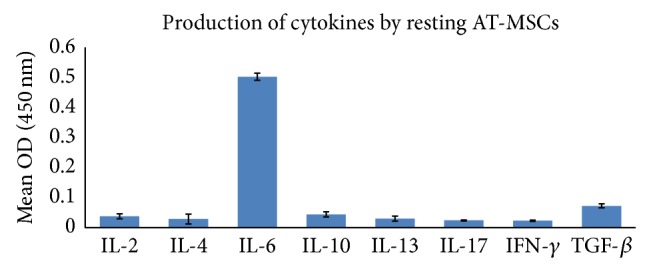
Cytokine profile secreted by AT-MSC in resting state. AT-MSC showed higher IL-6 and TGF-*β* production compared to other cytokines. Data presented the mean ± SD of five independent experiments. Resting AT-MSCs cytokine profile was used as blank to subtract background absorbance in coculture experiments for cytokine assays.

**Figure 4:**
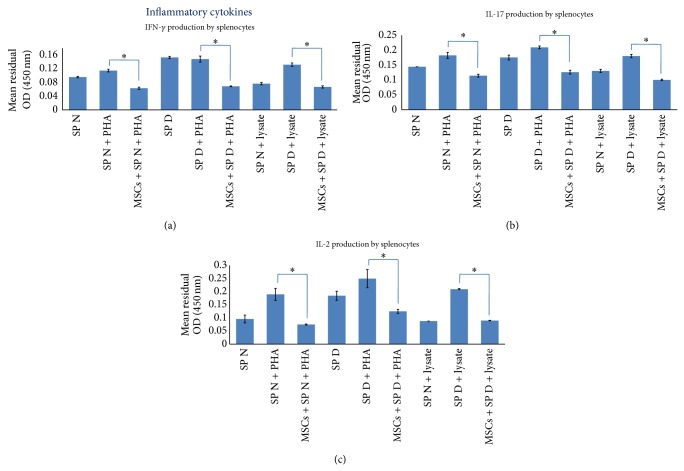
The immunomodulatory effects of AT-MSCs at passage 3 on inflammatory cytokines produced by splenocytes. Addition of AT-MSCs to islet, or PHA, stimulated splenocytes decreased IFN-*γ*, IL-2, and IL-17 production by splenocytes ((a), (b), and (c)). Background absorbance of cytokines produced by AT-MSCs was subtracted. Thus, the representative results show splenocytes related cytokines pattern. Data presented the mean ± SD of five independent experiments. ∗ indicates significant difference between groups (*P* < 0.05). SP N: normal splenocytes; SP D: diabetic splenocytes; PHA: phytohemagglutinin; lysate: pancreatic islet cells lysate.

**Figure 5:**
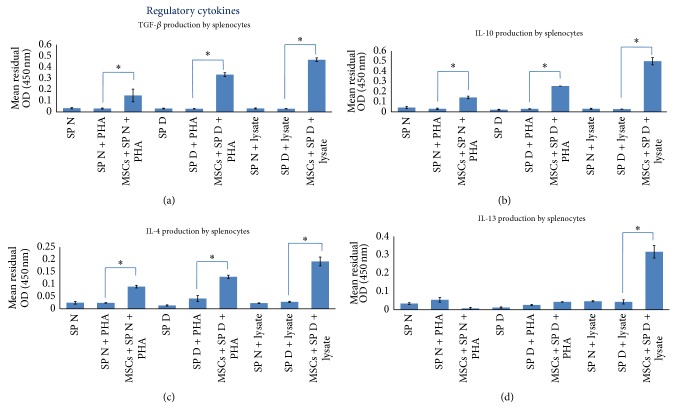
The immunomodulatory effects of AT-MSCs at passage 3 on regulatory cytokines produced by splenocytes. AT-MSCs in coculture with islet, or PHA, stimulated splenocytes increased TGF-*β*, IL-10, IL-4, and IL-13 production by splenocytes ((a), (b), (c), and (d)). Background absorbance of cytokines produced by AT-MCSs was subtracted. Therefore, the representative results show splenocytes related cytokines pattern. Data presented the mean ± SD of five independent experiments. ∗ indicates significant difference between groups (*P* < 0.05). SP N: normal splenocytes; SP D: diabetic splenocytes; PHA: phytohemagglutinin; lysate: pancreatic islet cells lysate.

To determine whether AT-MSCs modulate cytokine secretion by splenocytes, we prepared a coculture design with splenocytes supplemented by PHA or islet cells lysate. Analysis of cytokines production by resting AT-MSCs showed that they consistently secreted TGF-*β* and IL-6 in higher levels compared to other cytokines (Figure 3; *P* < 0.05). As previously described, AT-MSCs were used as blank to subtract background absorbance of their cytokines production. The results demonstrated that addition of AT-MSCs to lysate-stimulated diabetic splenocytes significantly decreased IFN-*γ*, IL-2, and IL-17 production by splenocytes (Figure 4; *P* < 0.05). The similar results were obtained when AT-MSCs were added to PHA-triggered splenocytes. In addition, this experiment indicated that AT-MSCs significantly increased TGF-*β*, IL-10, IL-4, and IL-13 production by splenocytes (Figure 5; *P* < 0.05). Although the increased production of these cytokines was also observed in the presence of PHA, increasing levels were significantly prominent in AT-MSCs cocultured with lysate-stimulated diabetic splenocytes, particularly for IL-13 (Figure 5).

### 3.7. Protective Effect of AT-MSCs on Insulin Secretion of Islets

**Figure 6:**
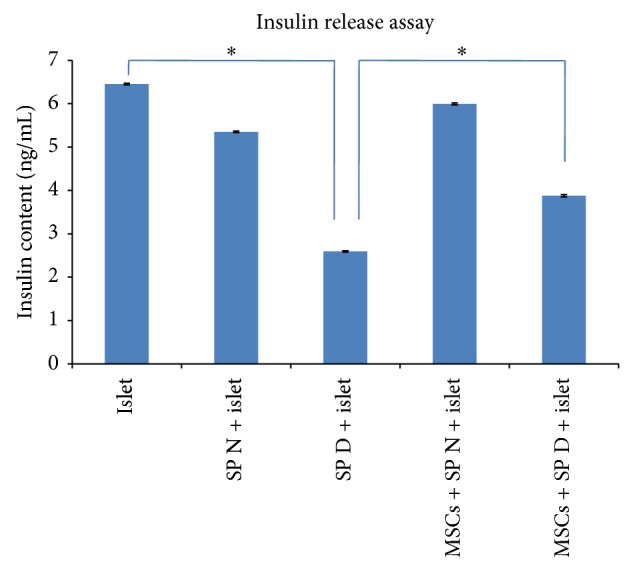
Pancreatic islet insulin release assay. Intact islets alone secreted distinct amount of insulin in response to stimulatory medium (6.45 ± 0.69 ng/mL). However, the insulin secretion levels decreased after coculture with diabetic splenocytes (2.59 ± 0.27 ng/mL). Interestingly, addition of AT-MSCs improved insulin secretion by injured islets (3.87 ± 0.41 ng/mL). Data presented the mean ± SD of five independent experiments. ∗ indicates significant difference between groups (*P* < 0.05). SP N: normal splenocytes; SP D: diabetic splenocytes; islet: mouse pancreatic islet.

To determine protective ability of AT-MSCs on islet cells function, islets were cocultured with reactive splenocytes and insulin content assay was performed. This experiment indicated that the crude islets alone secreted significant insulin levels when induced with stimulatory medium (6.45 ± 0.69 ng/mL). However, diabetic splenocytes significantly decreased insulin secretion by islets (2.59 ± 0.27 ng/mL; *P* < 0.05). In contrast, coculture with AT-MSCs led to a significant improvement in insulin secretion and the viability of islet cells (3.87 ± 0.41 ng/mL versus 2.59 ± 0.27 ng/mL; *P* < 0.05) (Figure 6).

## 4. Discussion

MSCs also referred to as adult multipotent stem cells represent a fibroblast-like morphology that have not only the capacity to differentiate into several tissues, but also have immunomodulatory and anti-inflammatory effects in vivo and in vitro [3, 21]. In this regard, previous studies have shown that MSCs induce peripheral tolerance and migrate to injured tissues, where they can inhibit release of proinflammatory cytokines and promote the survival of damaged cells [22–24]. It has also been reported that the underlying mechanism of MSCs effects might be attributed by both cell-cell contact [8] and soluble factors [9, 10].

Many studies that declared protective effects of MSCs on islet mass and differentiation potency to insulin-producing cells in vivo and in vitro collaborate for the application of MSCs regenerative role in T1DM [25–27]. Although many researchers have demonstrated the immunomodulatory activities of MSCs, in vitro potential assays able to predict or to correlate with therapeutic outcome of MSCs are still partially known. Thus, we assessedin vitro immunoregulatory effects of adipose-derived MSCs on autoimmune response in experimental diabetic model. In this respect, we aimed at three main goals: firstly, to evaluate suppressive effect of AT-MSCs on autoreactive splenocytes proliferation; secondly, to assess immunomodulatory effect of AT-MSCs on splenocytes cytokine patterns; and finally, to evaluate protective effect of AT-MSCs on functional islets. In the current study, MSCs were extracted from adipose tissue of healthy C57BL/6 mice and cultured to proliferate. Then, they were immunophenotyped and differentiated to osteocyte, adipocyte, and chondrocyte. Diabetic C57BL/6 mouse model was prepared by administration of consecutive low-doses of STZ and diabetic state was confirmed by serum glucose (>300 mg/dL), insulin levels, and pancreas histopathology. Pancreatic islets were isolated from normal mice and splenocytes prepared from normal and diabetic mice. To evaluate antiproliferative effect of MSCs, they were cocultured with diabetic splenocytes in the presence of islet lysate and proliferation assayed by MTT technique. The impact of MSCs on pancreatic islet function was assayed by insulin measurement in direct cocultures of diabetic splenocytes with intact islets. The effect of MSCs on cytokine patterns produced by splenocytes was assayed in cocultured splenocytes with MSCs in the presence of islet lysate for 72 hours. Inflammatory and regulatory cytokines were assayed in culture media by ELISA technique.

In our study, a STZ-induced diabetic model was prepared. Some strains of mice are Th1-dominant (C57BL/6, DBA/2, AKR, and CBA), but the others are Th2-dominant strains (BALB/c, BP2, and A/J) [28]. Many studies indicated that pathogenesis of T1DM is an autoimmune disease mediated by Th1-dependent inflammatory reaction [29, 30]. Therefore, in this study the selection of C57BL/6 mice as a suitable model for induction of autoimmune diabetes appears to be reasonable. High doses of STZ selectively kill the insulin-producing beta cells, whereas multiple low-doses of STZ induce expression of glutamic acid decarboxylase (GAD) autoantigen and develop an autoimmune response [31].

The shared immunophenotype and differential characteristics were identified in MSCs isolated from different tissues. Although some reports have shown that adipose-derived MSCs due to more simple isolation and expansion and also relative better immunomodulatory effects are a novel promise in experimental and clinical application [5, 32], isolated AT-MSCs had fibroblast-like morphology and differential capacity that were consistent with previous studies. In addition, analysis of cell surface markers on AT-MSCs showed a lack of hematopoietic markers, such as CD34 and CD45, and high expression of CD90 and CD29. However, some reports have shown that expression of surface markers on AT-MSCs varied in different strains of mice [33]. The biological characteristics similar to bone marrow mesenchymal stem cell (BM-MSCs) indicate that AT-MSCs may present an alternative source for future application [34, 35].

One of the main goals in this research was close to assessment of AT-MSCs suppressive effect on splenocytes proliferation in the presence of specific (islet cells lysate) and nonspecific (PHA) stimulators. The antiproliferative effects of AT-MSCs can be summarized as follows. (1) The results indicated that AT-MSCs in a ratio of 1 : 50, 1 : 250, and 1 : 1250 to splenocytes could inhibit stimulated splenocytes proliferation in a dose-dependent manner with the most inhibition at the lowest dilution tested (1 : 50; *P* < 0.05). However, AT-MSCs could induce cell death in some splenocytes in this concentration (1 : 50; *P* < 0.05). The similar effects of AT-MSCs were observed in the presence of islet lysate and PHA. In this respect, other studies have identified that mouse and human MSCs harvested from different tissues can suppress T lymphocyte proliferation stimulated by allogeneic cells or mitogens such as PHA [10, 11, 36, 37]. In a recent study, it has been shown that MSCs isolated from NOD and BALB/C mice could inhibit T CD4^+^ proliferation in full mismatch mixed lymphocyte reaction (MLR) assay. It has also been clarified that, in an autoimmune fashion in vitro, when MSCs are added to autoreactive T CD4^+^ and allogeneic dendritic cells in the presence of islet peptides, they could significantly suppress autoreactive T CD4^+^ proliferation [38]. We found that suppressive effects of MSCs were dependent on cell number. Several reports have demonstrated that immunosuppressive effects of MSCs occur in a dose-related manner in which it is considered independent of MHC interaction [39–41]. Although target cell-MSC interactions may be an important factor, the immunosuppressive effect of MSCs can also be mediated through the secretion of soluble molecules such as IL-10, TGF-*β*, IDO, NO, PGE-2, and HLA-G that are triggered following cross talk with target cells [4]. (2) AT-MSCs caused some cell death in resting splenocytes without each type of stimulators (*P* < 0.05). This may suggest that MSCs can induce cellular death through cell-cell contact as well as secretion of NO and IL-10 [9, 11]; also, they can drive splenocytes to produce IL-10 [11]. Although, in accordance with previous studies, we suggest that MSCs may induce cell death of splenocytes rather than cell cycle arrest [11], complementary studies are required to identify molecular mechanisms in detail. (3) In the absence of AT-MSCs, normal and diabetic splenocytes in response to nonspecific stimulator (PHA) showed similar fashion. In contrast, in response to islet lysate normal splenocytes were not responsive, whereas a sharp proliferation was obtained in diabetic splenocytes (*P* < 0.05).

In the next stage, we aimed at evaluating the effect of AT-MSCs on cytokine patterns produced by splenocytes. Analysis of cytokines production by AT-MSCs alone showed that they consistently secreted TGF-*β* and IL-6 in higher levels compared to other cytokines (*P* < 0.05). Several publications have confirmed spontaneous secretion of TGF-*β*1, IL-6, hepatocyte growth factor (HGF), HLA-G, and PGE-2 that associated with immunomodulatory properties of MSCs [42, 43]. Our in vitro experiment showed that AT-MSCs decreased production of IFN-*γ*, IL-2, and IL-17 and increased production of TGF-*β*, IL-4, IL-10, and IL-13 by stimulated splenocytes in response to islet lysate or PHA (*P* < 0.05). However, the increases in regulatory cytokines were significantly prominent in AT-MSCs cocultured with lysate-stimulated diabetic splenocytes, particularly for IL-13. Pathogenesis of many autoimmune diseases was mediated by several types of helper T lymphocytes (Th) such as Th1, Th2, and Th17 identified by secretion of distinct cytokine profiles [44]. Th1 cells produced IFN-*γ*, IL-2, and TNF-*α* and Th2 cells secreted IL-4, IL-5, IL-6, IL-10, and IL-13. Regulatory T cells and Th3 cells produce TGF-*β* and IL10 and another T helper subtype (Th17) produces IL-17. It has been demonstrated that MSCs have capacity to modulate immune reaction in vitro and in vivo, where they polarize proinflammatory state to anti-inflammatory state [3]. This is mediated by a shift in the Th1 to Th2 cell balance or by inhibition of Th17 differentiation and IL-17 production. Recent publications have reported the in vivo transplantation of MSCs derived from bone marrow and adipose tissue along with a therapeutic approach, where they switch host immune response towards a Th2 cytokine profile by downregulating Th1 cytokines and/or upregulating Th2 cytokines [22, 45–48].

Finally, in this study we used other approaches of MSCs properties to maintain in vitro viability and functionality of purified islets in the presence of reactive splenocytes. In this approach, it was found that AT-MSCs in coculture design had ability to provide islet functionality, proven by protected insulin secretion in coculture medium (*P* < 0.05). Several studies examined in vivo immunoregulatory effects of MSCs in cotransplantation with islets, which resulted in enhancing of islet allograft survival, insulin secretion, and sustained normoglycemia in STZ-induced diabetic model [43, 49, 50]. In a recent study performed by Karaoz et al. coculturing of normal and STZ-injured rat pancreatic islets with rat BM-MSCs has improved function and viability of islets [26]. They showed that cocultivation of STZ-injured islets and MSCs increased expression of IL-6 and TGF-*β*1 in the culture medium besides the expression of antiapoptotic genes. They also demonstrated that cytoprotective, anti-inflammatory, and antiapoptotic effects of MSCs were mediated through paracrine actions (spontaneous production of IL-6, TGF-*β*1, and HGF). Furthermore, effectiveness of MSCs in prevention and treatment of diabetes in NOD mice has also been reported [38]. The protective effects of MSCs may be dependent on both soluble mediators and also triggering of resident tissue precursor cells in vivo [17]. However, many studies declared that the regenerative role of MSCs could be mediated by differentiation potential to insulin-secreting cells in vivo and in vitro [18, 25].

In brief, we have demonstrated that AT-MSCs in a dose-dependent manner inhibit splenocytes proliferative response to specific and nonspecific triggers that may be mediated by both cell-cell contact and soluble mediators such as TGF-*β*1 and IL-10. AT-MSCs can also polarize cytokine patterns produced by splenocytes toward a regulatory pattern. Finally, our results indicated that AT-MSCs through cytoprotective and anti-inflammatory effects can effectively sustain viability as well as insulin secretion potential of islet cells in the presence of reactive splenocytes. Although we note that adjunct in vivo studies are required to completely investigate the immunomodulatory functions of MSCs in autoimmune diseases, this study may provide some basic information regarding application of human MSCs in treating type 1 diabetes.

## Supplementary Material

A figure illustrating AT-MSCs characterization.

## Figures and Tables

**Table 1:** Test and control groups in coculture experiments.

Control groups		Test groups	
Normal mice	Diabetic mice	Normal mice	Diabetic mice
SP N	SP D	SP N + lysate	SP D + lysate
SP N + PHA	SP D + PHA	SP N + lysate + MSCs	SP D + lysate + MSCs
SP N + MSCs	SP D + MSCs		
SP N + PHA + MSCs	SP D + PHA + MSCs		
AT-MSCs			

SP N: normal splenocytes; SP D: diabetic splenocytes; PHA: phytohemagglutinin; lysate: pancreatic islet cells lysate; AT-MCSs: adipose tissue-derived mesenchymal stem cells. SP and AT-MSCs monocultures were used as blank in experiments.
