# Supplementary material for: Adipose Tissue-Derived Mesenchymal Stem Cells Exert In Vitro Immunomodulatory and Beta Cell Protective Functions in Streptozotocin-Induced Diabetic Mice Model
Source: J Diabetes Res. 2015 Mar 29;2015:878535. doi: 10.1155/2015/878535 (PMC4393922; doi:10.1155/2015/878535)
Supplement: Supplementary file 1 — A figure illustrating AT-MSCs characterization. [file 878535.f1.docx]

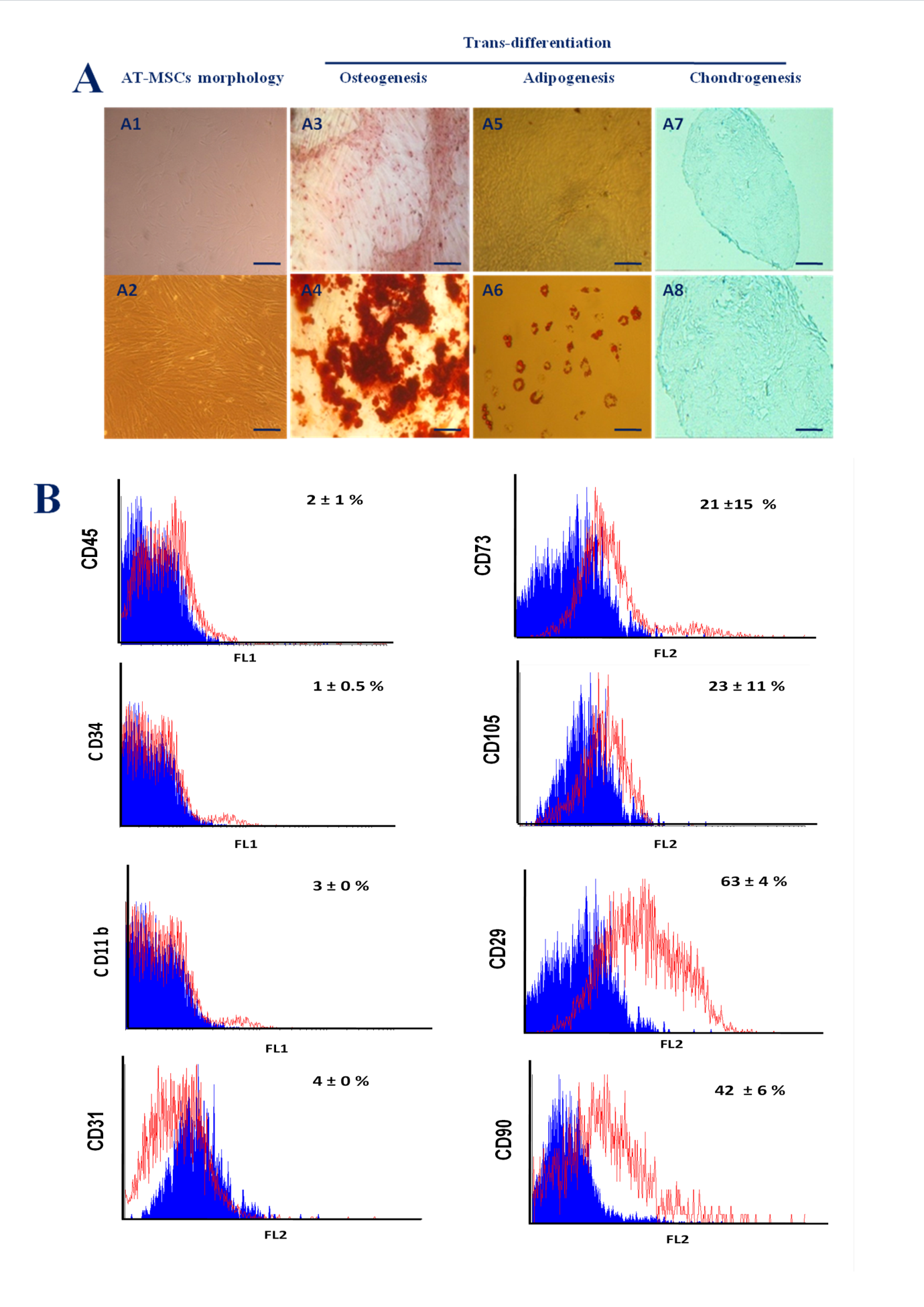


Morphology and plasticity of AT-MSCs isolated from C57BL/6 mice **(A)**. After 6-8 days, the fibroblastoid adherent cells developed the onset of colony formation (A1). After 10-12 days, Single cell-derived clones were expanded and gradually reached confluency with a whirlpool-like array (A2). Plasticity of AT-MSCs examined by differentiation of cells into osteogenic, adipogenic, and chondrogenic lineages in the presence of appropriate induction media. After 21 days, osteogenesis was confirmed by calcium deposition in the matrix visualized with alizarin red staining (A4). Lipid droplets were detectable by oil red O staining after 21 days of adipocytic induction (A6). After 21 days of induction, chondrogenic differentiation of AT-MSCs was achieved; more than 80% of all cells stained positively with alcian blue (A8). Control cultures in normal growth mediums were also maintained in parallel (A3, A5, and A7). Scale bars represent: A1-A7 = 100 μm; A8 = 400 μm. Flow cytometric analysis of surface expression for phenotypic markers in isolated AT-MSCs **(B)**. Isotype controls are presented as blue histograms and analyzed markers as red histograms. Data demonstrates AT-MSCs are negative for CD11b, CD34, CD45, and CD31 whereas they were positive with different percentages for CD73, CD90, CD105, and CD29. The figure shows one representative result from five independent experiments.
